# Research-policy partnerships - experiences of the Mental Health and Poverty Project in Ghana, South Africa, Uganda and Zambia

**DOI:** 10.1186/1478-4505-10-30

**Published:** 2012-09-14

**Authors:** Tolib N Mirzoev, Maye A Omar, Andrew T Green, Philippa K Bird, Crick Lund, Angela Ofori-Atta, Victor Doku

**Affiliations:** 1 Nuffield Centre for International Health and Development, Leeds Institute of Health Sciences, University of Leeds, Charles Thackrah Building, 101 Clarendon Road, Leeds, LS2 9LJ, United Kingdom; 2 Alan J Flisher Centre for Public Mental Health, Department of Psychiatry and Mental Health, University of Cape Town, 46 Sawkins Road, Rondebosch, 7700, South Africa; 3 University of Ghana Medical School, P.O.BOX 3859, Accra, Ghana; 4 Kintampo Health Research Centre, P.O.Box 200, Kintampo, Brong-Ahafo Region, Ghana

**Keywords:** Partnership, Mental health, Africa, Research-policy, Ministry of health

## Abstract

**Background:**

Partnerships are increasingly common in conducting research. However, there is little published evidence about processes in research-policy partnerships in different contexts. This paper contributes to filling this gap by analysing experiences of research-policy partnerships between Ministries of Health and research organisations for the implementation of the Mental Health and Poverty Project in Ghana, South Africa, Uganda and Zambia.

**Methods:**

A conceptual framework for understanding and assessing research-policy partnerships was developed and guided this study. The data collection methods for this qualitative study included semi-structured interviews with Ministry of Health Partners (MOHPs) and Research Partners (RPs) in each country.

**Results:**

The term partnership was perceived by the partners as a collaboration involving mutually-agreed goals and objectives. The principles of trust, openness, equality and mutual respect were identified as constituting the core of partnerships. The MOHPs and RPs had clearly defined roles, with the MOHPs largely providing political support and RPs leading the research agenda. Different influences affected partnerships. At the individual level, personal relationships and ability to compromise within partnerships were seen as important. At the organisational level, the main influences included the degree of formalisation of roles and responsibilities and the internal structures and procedures affecting decision-making. At the contextual level, political environment and the degree of health system decentralisation affected partnerships.

**Conclusions:**

Several lessons can be learned from these experiences. Taking account of influences on the partnership at individual, organisation and contextual/system levels can increase its effectiveness. A common understanding of mutually-agreed goals and objectives of the partnership is essential. It is important to give attention to the processes of initiating and maintaining partnerships, based on clear roles, responsibilities and commitment of parties at different levels. Although partnerships are often established for a specific purpose, such as carrying out a particular project, the effects of partnership go beyond a particular initiative.

## Introduction

Partnerships are increasingly common in conducting research projects [1,2] at different levels from international collaborations [3,4] through to individual programmes such as tuberculosis control or mental health [5-7]. Although literature exists examining the impacts of different partnerships [8,9], there is a lack of clarity about the nature of their processes and potential benefits, in different contexts for different stakeholders such as policy-makers and academic/research institutions. This paper makes a contribution to filling this gap.

A partnership arrangement was established for the Mental Health and Poverty Project (MHaPP), implemented in four countries (Ghana, South Africa, Uganda and Zambia) between 2005–2010. The MHaPP purpose was to develop, implement and evaluate mental health policy in these countries [10]. The MHaPP project was initiated by the Consortium Coordinator (University of Cape Town) and implemented by a consortium of 14 institutions from Africa and Europe, including Ministry of Health (MOH) representatives from four countries. It considered partnership between research institutions and MOHs important to influence policy and service delivery changes. It was planned that MOH and country research teams would work together to enhance the potential for evidence-informed policy and to ensure the sustainability of the collaborative research programme.

This paper aims to analyse the experiences of partnerships between the MOH partners (MOHPs) and Research Partners (RPs) within the four MHaPP countries. The specific objectives of this paper are to: a) develop a conceptual framework for understanding and assessing research-policy partnerships, based on the literature; b) analyse experiences of research-policy partnerships established within the MHaPP project, using a conceptual framework and c) to identify implications for setting and maintaining effective research-policy partnerships. We focus primarily on issues related to the *processes* of partnerships and, although we recognise the inevitable overlaps with wider effects of partnerships such as research quality and the use of knowledge, we do not aim to cover in detail the *contents* of MHaPP research.

### What are Partnerships?

The notion of collaborations between researchers and non-researchers in conducting research is not new. For example, in 1983 Henkel and Kogan referred to numerous benefits of collaborative work between researchers and policy-makers [11]. These were similar to the four key motivations of those engaged in collaborative research reported by Denis and Lomas two decades later: broadening the range of choices in defining the problems, better interpretation of research findings, greater practical use of research findings and bring about change in the way researchers think and practitioners work [12].

Partnerships, one specific form of collaborative research, vary along a spectrum of formality of interrelationships, from loose relationships between agencies working towards a commonly defined set of objectives through to agreements (possibly legal) on specific elements of relationships such as communication [13,14] or use of resources. Depending on their composition and purpose, health-related partnerships may include: public-private [5,15], service provider-patient interactions [16], inter-sectoral [17], academia-industry [18,19], country-level financing or aid coordination [20-23], thematic [24-26] and global or international such as The Global Fund to Fight AIDS, TB and Malaria. These are not mutually exclusive and, for example, Sub-Saharan Africa has been the geographical focus of thematic International-level Health Partnership [3,27].

In this study a partnership is defined as a collaborative arrangement, agreed between the parties (in this case, MOH and researchers in Ghana, South Africa, Uganda and Zambia), established to achieve common objectives and with agreed roles and inputs from the parties.

#### Understanding research-policy partnerships

Three broad types of research-policy partnerships, sometimes defined as ‘*research-decision-maker partnerships’*[28], can be distinguished:

1. *Researchers - policy-makers (individuals and organisations involved in setting health policies, typically national MOH and other government officials) partnerships* concerned with Getting Research Into Policy and Practice (GRIPP) and affected by the perceived information needs as well as priority-setting and funding for research [28-30].

2. *Researchers - public partnerships,* where relationships are set up with communities as a way of influencing policy decisions. Partners contribute their expertise to enhance understanding of given phenomena and to integrate the knowledge gained with action to benefit the community involved [31,32].

3. *International partnerships between researchers* as a way of enhancing researchers’ credibility and power to affect policy decisions often in specific fields such as policy research, clinical studies or operational research. The principles of an equal research partnership in setting agendas and implementing international projects often need monitoring [33,34] to ensure local research needs in the study countries are addressed and capacity of Southern institutions is sustainably strengthened.

The above partnership types are not mutually exclusive and different combinations may exist within a single partnership arrangement that can be established for conducting health research as well as translating it into policy and practice. Indeed, the MHaPP project is an example of the first (within individual countries) as well as the third (at Consortium level). In this paper when we refer to research-policy partnerships we focus on the first type (research – policy-maker) though recognise that the two levels are related.

There has been a shift over the last twenty years in research-policy partnership models from the ‘two communities’ model to the ‘linkage and exchange’ model, sometimes also referred to as the network approach [35]. In the former the researchers and policy-makers are motivated by different agendas and operate in their discreet worlds and intermediaries - often knowledge brokers – are required to bridge the link between these two communities [35-37]. In the linkage and exchange model the two groups (researchers and policy-makers) are perceived as members of a policy network with a potential for direct links between the two groups through, for example, joint identification of research priorities, decision-makers’ involvement in research processes and researchers’ involvement in decision processes [35,38,39].

#### The processes of research-policy partnerships

While there is much literature on different types and elements of partnerships [2,4,8,9,18,28,40,41], there is less research on the dynamics or *processes* of relationships between the different parties within research-policy partnerships. Differing approaches to partnerships in general can be distinguished in the literature, ranging from competition through to cooperation [42]. Within the latter, different interactions can be identified: cooperation, collaboration and coordination [43], with the last being the most comprehensive form potentially involving joint planning and implementation of activities and sharing of resources.

The four stages of Tuckman’s team-building concept (forming i.e. when teams are formed; storming i.e. when roles are clarified and changes are made as necessary; norming i.e. when key agreements are reached on the roles and responsibilities; and performing i.e. when the team functions effectively) [44] can be applied to partnership processes with some modifications that we propose as follows.

We distinguish the following three broad phases of partnership processes:

• *Phase 1: Establishing the partnership* (comprising the forming, storming and norming stages of Tuckman’s taxonomy) – identification of objectives, potential advantages and disadvantages of partnerships, agreement on the ‘rules of the game’ including modes of communication and, where applicable, accountability and joint reviews;

• *Phase 2: Supporting and performing* (the performing stages) – where the partnership starts to deliver expected outputs or, depending on the objectives, contributes towards improved processes within and between the organisations;

• *Phase 3: Dissolving partnerships* – although many partnerships are seen as long-term relationships they may be dissolved after achievement of objectives or following the completion of a programme. Some partnerships, such as Research Consortium arrangements, may be re-grouped for the next research project.

The above seemingly linear process can include iterations within and between the stages (for example, inadequate performance leads to changes in working arrangements such as approaches to communication). Furthermore, there is no set length of time for each phase and the efficiency of the process may depend on the previous working relationships between the parties, degree of similarity between the organisational and institutional approaches and procedures and other factors.

Some studies explore the roles of researchers and policy-makers in research-policy partnerships. The researchers’ role is usually confined to conducting research and communicating the new knowledge [28,35]. Ross et al. proposed three models of decision-maker involvement in research processes: formal supporter, responsive audience and integral partner; the last being the most active involvement of decision-makers in the research processes [45]. However, there is less systematic assessment of partnership processes. Building on the existing literature, further in this paper we propose a conceptual framework to help understand and assess processes of research-policy partnerships as a primary focus of this study.

#### Key influences on effectiveness of research-policy partnerships

Different influences appear to affect the success of research-policy partnerships in achieving its objectives, which can be summarised in the following four groups:

1. Clarity of overall purpose of a partnership and secured political legitimacy where appropriate [40,46].

2. The existence of a long term relationship, often progressing from pre-existing working relationships providing a platform for clear expectations and roles of different partners, throughout the partnership process [45-47].

3. An appropriate, often flexible, administrative structure and clear procedures to ensure trust, equivalency between partners (for example in decision-making), effective communication and conflict resolution as needed [13,39-41,46].

4. Mechanisms for establishing and maintaining collaborative capacity and maintaining objectivity and excellence of research within the context of the different values and agendas (including vested interests) of partners [40,48].

A caveat is appropriate here. Different terms are used interchangeably in the literature on partnerships. For example, the different partnership models (synergy, transformation and budget enlargement) described by Mackintosh [13] can also be seen as the set of *objectives* for, or intended *outcomes* of, partnerships. Similarly, the eight *prerequisites* of *facilitating factors* for effective partnerships suggested by Balloch and Taylor [40] can also be seen as *characteristics* of well-functioning partnerships or objectives for capacity development as part of partnership arrangements.

### Setting the Context: Ghana, South Africa, Uganda and Zambia

All four countries in MHaPP have a wide range of competing health challenges [49]. Table 1 summarises the key contextual issues in the four countries, focusing on mental health.

**Table 1:** Context of Ghana, South Africa, Uganda and Zambia

	**Ghana**	**South Africa**	**Uganda**	**Zambia**
Total health expenditure as % of GDP	7.8	8.2	8.4	5.9
Per capita total health expenditure (PPP Int $)	114	843	112	80
% of health budget allocated to Mental health	0.5	2.7	0.7	0.4
Psychiatric nurses per 100,000	2	7.5	2	5
Psychiatrists per 100,000	0.08	1.2	1.6	0.02
Form of health system decentralisation	Centralised implementation, some delegation to regions	Devolution to provinces	Devolution to districts	Deconcentration to regions and district health authorities
Current status of mental health policy: year of adoption and implementation	2000	1997 (draft)	2000 (draft)	2005
	Patchy implementation	Inconsistent implementation, on-going revision of policy	Moderate implementation, ongoing revision of policy	Plans recently developed, poor implementation

Sources: [50-52].

The countries have different health system structures and financing mechanisms though they share the challenge of low resource levels with health expenditure ranging from 5.9% of GDP in Zambia to 8.4% in Uganda [50-52]. Mental health remains a low priority as a policy issue [49] and mental health services are provided largely by the public sector [50,53-55].

Only Ghana and Zambia had an approved mental health policy, in 2000 and 2005 respectively, though in Zambia no implementation plans had been yet developed which resulted in poor implementation. In South Africa policy guidelines were approved in 1997 which provided a framework for implementation, even though the draft policy was still not formally adopted [56]. The situation in Uganda was similar, with implementation of a draft policy since 2000 [50].

### Setting the Context: the MHaPP project

The choice of countries for the MHaPP was driven by a) differences in their contexts and the resultant knowledge being potentially applicable to a range of other developing countries and b) existence of positive relationships between the research teams and the MOH officials providing an opportunity to influence policy processes [10]. The MHaPP research included two Phases: Phase 1, situational analysis (Years 1 and 2) which aimed to understand the context and identify priority areas in each country; and Phase 2, implementation and evaluation of the following country-specific interventions (Years 3–5): Mental Health Policy and Legislation (all countries except Zambia), Mental Health Information Systems (Ghana and South Africa) and Strengthening District Mental Health Service Delivery (all countries). The details of the overall design of MHaPP, results of situational analyses and lessons learned from different interventions are published elsewhere [10,50,53-56].

In Ghana, Uganda and Zambia one research organisation was involved in partnerships with MOH. Although two research organisations were involved in the MHaPP project in South Africa, the partnership with the MOH involved primarily the Consortium Coordinator.

Different African and European institutions were the lead partners on thematic and methodological issues which cut across all the four study countries (for example, intervention design during the Phase 2, capacity strengthening and knowledge communication). A degree of camaraderie was established between these lead partners and their country collaborators as they shared experiences (development of data collection tools, data collection, analysis and writing) in working on the lead partners areas of expertise and interest. RPs in the four African countries conducted data collection and analysis, supported by lead partners. MOHPs in each African country contributed to research objectives and design and supported data collection and analysis, and played a role in knowledge transfer.

The partnerships were formalised in all countries through a Memorandum of Understanding or Letter of Support (see Table 2). The template for the above memoranda was provided by the Consortium Coordinator, typically covering roles and responsibilities of parties, processes for staff recruitment and financial accountability, communication mechanisms and key principles for decision-making including mechanisms for conflict resolution. The key principles and mechanisms for the above included joint responsibility for the project though the ultimate responsibility of RPs for the research, inclusive decision-making and, where appropriate, mediation by the Consortium Coordinator.

**Table 2:** Key partnership issues within the four MHaPP countries

**Key issue**	**Ghana**	**South Africa**	**Uganda**	**Zambia**
Partners involved				
MOHP	Chief Psychiatrist in Ghana Health Service	Directorate of Mental Health and Substance Abuse, national Department of Health	MOH, Mental Health Unit	MOH, Mental Health Unit
RP	Kintampo Health Research Centre and University of Ghana Medical School	Department of Psychiatry and Mental Health, University of Cape Town	Department of Psychiatry, Makerere University	Departments of Psychiatry, and Social Development Studies, University of Zambia
Degree of formalisation	Memorandum of understanding. Support Letter from the Director General of the Ghana Health Service	MOH letter of support	Memorandum of Understanding	Memorandum of Understanding
Decision-making in the project, including operational (e.g. staff recruitment, budget management) and strategic (study design)	Mostly RP (operational decisions), some consultation with MOHP on strategic issues	Mostly RP (operational decisions), some consultation with MOHP on strategic issues	Joint decision-making covering both operational and strategic issues	Both operational and strategic issues - mostly at PI discretion at initial stages; in consultation with MOHP after the change of PI
Other partnership characteristics	Two Principal Investigators (PIs) from RP	Research team spread across the three sites (including two provinces). The RP was also MHaPP coordinator	Long-standing relationships between RPs and MOHPs	Substantial changes to the composition of partners were made during the project, including change of PIs

## Methods

This paper reports findings from a qualitative study on research-policy partnerships established in the MHaPP project. It examines research-policy partnerships within the four African study countries only and we do not cover other levels of partnerships in MHaPP such as North–south collaboration or relations between research teams in the different countries.

The development of the conceptual framework was informed by a review of published literature, in English, identified through PubMed and Medline databases using combinations of the following keywords: ‘partnerships’, ‘research-policy’, ‘partnership processes’, ‘partnership framework’. In addition, a search of grey literature was conducted using ELDIS (Electronic Development and Environment Information System) gateway, covering development policy, practice and research.

We collected data in November 2009 using 11 semi-structured interviews with respondents from both the MOH (n = 5) and the research teams (n = 6) in the four project countries. Purposive sampling, a common approach in qualitative studies, was used in selecting the respondents. The University of Leeds team selected respondents who had been the lead individuals in each partner organisation and thus had detailed knowledge of partnership processes. Where two people had played a leading role within a partner organisation, we interviewed both to ensure the representation of multiple viewpoints in understanding the partnership processes. We distinguish between ‘Research partners’ (RPs) and ‘MOH partners’ (MOHPs) involved in this partnership. The views of the latter may differ from those of the MOH as an institution.

The interviews were conducted by the University of Leeds team, using separate interview guides for MOHPs and RPs. The interview guides included broad, flexible questions covering the different components of the conceptual framework (described in the next section) and were adapted for each respondent as appropriate. The study conformed with the University of Leeds ethical standards applicable to qualitative studies. Guarantees of privacy and confidentiality were given to respondents who gave informed consent. The respondents did not receive any rewards for participation in this study. The interviews were recorded and transcribed. Analysis was performed by the University of Leeds team using an adaptation of the Framework Approach, which included the following stages: familiarisation with the data, identifying a thematic framework through coding of data to reflect the aims of the study and what is emerging from the data, arranging data using the thematic framework, interpretation of data to look for patterns and associations in the data, and developing subsequent explanations as appropriate [57]. Responses were triangulated between the two groups of respondents (MOHPs and RPs) and compared across the countries and with the literature.

Data collection and analysis were conducted by University of Leeds researchers who were, as a project partner, familiar with the project, but were not directly involved in country-level MOHP-RP partnerships. Usually study respondents, as subjects of research, are not authors of research papers. The initial intention - given the participatory nature of this study and in the spirit of partnership - was for all partners to be represented as co-authors on this paper. However, this was not achievable because the time pressures prevented adequate contributions from all partners to comply with the general requirements for co-authorship to academic publications.

### Findings

Our study is guided by the conceptual framework, which is presented next, followed by identification of key findings, structured by the components of the conceptual framework.

### Conceptual framework

**Figure 1:**
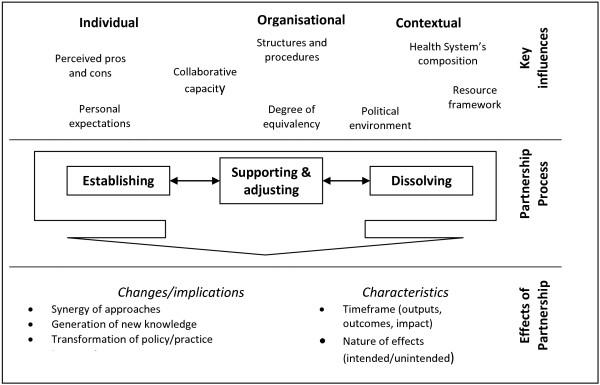
**Conceptual framework of research-policy partnerships.** Based on: [2,28,40,45].

Figure 1 sets out our conceptual framework for *research-policy partnerships* which draws on the existing literature on the subject and guides our analysis.

It distinguishes three components. The **process** of partnership, drawing primarily on the stages of team-building concept [44], is placed in the centre of the framework representing the primary focus of our study, and includes: *establishing*, *supporting/adjusting* and *dissolving* the partnership. Each phase may include specific steps. For example, establishing the partnership may include informal discussions followed by formalisation of agreed arrangements in a memorandum of understanding; supporting partnership may include on-going communication and adequate distribution of tasks; and dissolving partnership may include agreements on distribution of access rights to any assets or knowledge generated by the partnership. Iterations may also be possible between the stages – for example, the process of supporting the partnership may lead to further agreements/adjustments such as communication approaches or adjustment of the parties’ roles.

Different **key influences** affect the success of the partnership; these can be categorised across the three levels: *individual* (such as personal expectations and perceived benefits) [45-47], *organisational* (such as organisations’ governance structures and decision approval procedures and relative roles in partnership) [13,39-41,46] and *contextual* (political environment or composition of wider health system) [28,36,45]. Each may enable or constrain the partnership. They include influences from both within the partnership (e.g. communication approaches or partners’ collaborative capacity (complementarity of partners’ abilities to perform tasks)) and beyond (policy issues, or availability of external resources such as financial or human resources). The factors are interrelated (for example, collaborative capacity is affected by structures and procedures).

**Effects** of partnership are multiple and may be intended or unintended. Intended effects may include successful completion of the research project; unintended effects could be changes to an organisation’s decision-making processes. Effects may be positive or negative and within the partner institutions/networks and beyond [4]. They can include transformation of policy/practice, increase in resources and synergy of partners’ working approaches [28-32]. It is important to distinguish the effects of partnership (as a process) from the effects of research on policy and practice; this paper focuses on the former though recognises that the two are related.

We now report findings structured according to the three components of the conceptual framework, followed by identification of lessons learned from the partnership experiences.

### The concept of partnership

The term partnership was interpreted by most respondents as involving mutually-agreed goals and objectives. The principles of trust, openness, equality and mutual respect were identified by respondents as constituting the core of partnerships, as shown by a typical description of partnership provided by one RP:

"…reciprocal relationship, where there is openness, and where we can all share in a way where we are in some sort of equality."

Partnership was perceived as being more than just collaboration. Partnership was perceived as being a formalised relationship – as illustrated by the existence of a written partnership agreement such as a Memorandum of Understanding - with agreed principles of pooling resources and skills and reduced independence.

The specific partnership arrangements differed across the four countries. RPs were academic institutions in all countries, but there were variations in the policymaking partner. In South Africa, the MOHP was the Directorate of Mental Health and Substance Abuse in the national Department of Health. In Uganda and Zambia the partnership was with the respective MOH Mental Health Units whereas in Ghana the partnership involved the government Chief Psychiatrist based in a tertiary hospital within the Ghana Health Service.

The primary MHaPP project objective was to contribute towards improved national mental health policies and services [10] and the partnership was seen as a means of achieving this. Respondents’ interpretations of partnership objectives also revealed individual motives for entering into, and expectations from, partnerships. These included ‘*improving research skills*’ by the MOH from South Africa, Ghana and Uganda and ‘*establishing and maintaining an effective dialogue* [with the MOH]’ by the RPs in South Africa. No reference was made by respondents to monetary benefits (such as salaries) from the partnership as a perceived expectation from the partnerships. All these expectations can be interpreted as factors that affected the functioning of the partnership, as explored later.

### Partnership processes

Respondents were asked what stages they could conceptually distinguish in partnerships. Most respondents identified two broad phases - the ‘initiation’ or ‘start’ of the partnership and its subsequent ‘functioning’ with no references to the third potential phase of ‘dissolving’ of the partnership (which perhaps reflects the stage of the project at the time the interviews were conducted).

The initiation of the partnership was largely done at a personal level through existing contacts between RPs and MOHPs. A partner within each country was initially approached by the Consortium Coordinator (RP in South Africa) and then subsequently approached another partner. In Uganda the Coordinator approached RP first whereas in Zambia the initial contact was made through the MOH. The role of the World Health Organisation country office as a catalyst of initial partnership contact was emphasised in Ghana whereas in all other countries the contacts were made directly between the partners.

In Ghana and Zambia the RP’s initial contact was with the top-level MOH leadership whereas in the other two countries it was with the Mental Health Unit. However, subsequent day-to-day interaction was done with the relevant MOH Unit/Department in all countries.

No references were made by respondents to the roles of other actors at national (e.g. civil society) or international (e.g. global partners in the research consortium) levels in initiating and functioning of partnerships.

The MOHPs and RPs had clearly defined partnership roles. The MOHP’s role was perceived primarily as providing political support to the research process or ‘*unlocking doors*’, although some respondents believed that MOHPs played a more central role in research conceptualisation (e.g. setting the research agenda) and implementation (e.g. validation of findings and facilitating their transfer into policy and practice). This reflected what had been identified in the Memoranda of Understanding. The research agenda was led primarily by RPs in Zambia and South Africa though references were made in Uganda to joint agenda-setting, as per the Memorandum of Understanding. In some countries, the MOHPs’ critical (research) ability appears to have been overshadowed by the perceived academic rigour of RPs, thus potentially contributing to lesser involvement of MOHPs in research processes. As one MOHP commented:

"it was a bit threatening, you know, coming from the technical field and going to interact with academics and researchers***…***then you are not sure if you have enough skills at that level… the background that you have in research, is it going to be adequate?"

The RP’s involvement in policy processes differed between countries. Experiences ranged from close engagement and consultation in Uganda and Ghana through to evidence elsewhere of a clear labelling of MOH as *the* policy-maker, as reflected by one RP:

"we were told quite firmly… that we were one of… 60 odd research entities or NGO’s or stakeholders that they could consult with if they chose to, but they are …[Ministry of Health]… and they have a mandate to develop policy and it was not our role…"

"Some RPs felt that MOHPs “maybe… felt threatened… if we were perhaps taking over what they perceived to be their mandate in policy development”."

No references were made by the respondents to the leadership styles and approaches and their implications on the roles of MOHPs and RPs within the partnerships.

Communication between RPs and MOHPs was generally perceived to be effective though the degree of continuity and modes of communication differed. In Uganda, communication included both formal (e.g. letters) and informal (e.g. phone calls) types; in South Africa formal types of communication prevailed at the initial stages of the project. In Ghana and South Africa, in contrast with the other countries, the communication involved additional agencies (Provincial Department of Health in South Africa and District Administration of research site in Ghana), with resultant negative implications for continuity and efficiency.

In Zambia, in contrast to the other countries, the partnership process experienced difficulties such as, for an initial period, deteriorating trust and lack of continuous communication. As a result, a new RP and additional MOH focal person were brought in, facilitated by the Consortium Coordinator and this may have contributed to improved communication and coordination between the MOHP and RP at later stages of MHaPP. This different experience may reflect the fact that other countries (for example, Uganda) already had well-functioning relationships between the MOHPs and RPs prior to the project. Although not evident in the data, the fact that partnership was not dissolved as a result of difficulties may suggest that partnership in Zambia became stronger as a result of these experiences.

### Key influences on effectiveness of partnerships

Various influences were identified for the effectiveness of partnerships. Some overlap (for example, communication approaches) and the three groups of key influences identified in the conceptual framework are inter-related (for example, contextual influences determine the degree of equivalency of different organisations).

#### Individual influences

Individual or personal/personality-related influences were referred to by all respondents as important. Within this, different issues were raised.

Both the MOHPs and RPs felt that effective personal relationships founded on trust and mutual respect are crucial for effective functioning of partnerships of this complex nature. Personal relationships were seen as important in communication between RPs, the (national-level) MOHPs and the sub-national research sites (for example, provinces in South Africa or Kintampo District in Ghana). The importance of personal relationships was particularly emphasised in Ghana, where the arrangement to have two Principal Investigators (PIs) (one based in-country and another abroad) added to the complexity of relationships, and in Uganda, where mutual respect and effective personal relationships between the RP and MOHP contributed to the successful partnership processes.

The ability to compromise (both by MOHPs and RPs) was also perceived as important in ensuring effectiveness of partnership processes. An example was the MOHP’s willingness to accept critical situational analysis reports in Uganda and Ghana, which assessed the state of mental health in the light of existing policy and services in each country. Another example, raised by RPs was their ability to compromise with the research agenda such as selection of thematic areas for Phase 2 interventions. One RP described this as “*…working out when it is time to not fight a battle you cannot win…*”.

Previous experiences and links appear important in establishing and maintaining partnerships in all countries. In all countries, except Zambia, RPs had previous experiences of interacting with the MOH, either as teacher-student or as previous partners. In addition, in one country the PI was a former member of the government and had a comprehensive understanding of policy processes and other government procedures.

Both MOHPs and RPs perceived opportunities and potential threats from the partnerships; on balance the former were seen as prevailing at both individual and organisational levels. The RPs were seeking ways to facilitate the research process: *“…channels that our research officer could go in and out and get his work done*”. The MOHPs (in South Africa and Zambia) expected improved research skills; and the possibility of involvement in publications was emphasised by Ugandan and Ghanaian MOHPs. Two perceived individual threats from the partnership were raised by the South African and Zambian MOHPs. These were: feeling threatened and perhaps disempowered by their lack of academic skills, which may have affected the degree of their involvement in research processes; and fear of disclosure of information, originating from a lack of clarity as to how project findings would be used.

#### Organisational influences

The individual level issues were closely related to organisational ones. This was particularly marked in Uganda, where the Mental Health Unit comprises one person, and in Ghana, where there is no specific MOH Mental Health Unit and policy is dealt with by the Chief Psychiatrist based in a tertiary hospital. Two organisational factors affected partnerships in MHaPP: the degree of formalisation of roles and responsibilities and the internal structures and procedures.

The degree of formalisation of roles and responsibilities within the partnership was perceived as the most important factor, as reflected by one MOHP:

"it must be clarified in this partnership, how do we define the relationship, the boundaries and clarify who is going to do what, agreeing in the first place and becoming realistic"

This formalisation of relationships was achieved through either a letter of support or Memorandum of Understanding. The division of roles appears to have taken account of collaborative capacity with RPs being responsible primarily for carrying out the research project and the MOHPs advising and providing political support.

The degree of transparency and the related degree of joint decision-making by both parties was seen as important for the MOHP to feel ownership for the project. However country experiences in relation to joint decision-making differed. Joint project-related decisions were reported from one country whereas, in another, the RP recruited and dismissed research staff without consulting the MOHP.

The mechanisms for conflict resolution within partnerships were raised by the study respondents as being important in ensuring effectiveness of partnerships. The need for clear mechanisms for conflict resolution was raised in all countries and was particularly emphasised in relation to the case of Zambia, where the partnership experienced difficulties, perhaps due to a lack of previous partnership arrangements.

The internal organisational structures and procedures affected the degree of effectiveness of the partnership. The relative power of the Mental Health Unit within the wider MOH was seen as an important consideration in committing the MOHPs to various partnership tasks:

"the level of decision-making that we had… because, for example, I don’t have the power in this meeting to decide that the department will do this, even my director doesn’t have the power…"

The communication rules were important in affecting the efficiency and effectiveness of dialogue within the partnership. Continuous monitoring and prompt reaction to emerging issues were particularly emphasised in Ghana and South Africa, countries where Provincial and District authorities played significant roles in project implementation particularly during Phase 2 of the project (implementation and evaluation of interventions).

#### Wider contextual influences

Respondents highlighted two contextual factors affecting partnerships: the political environment and the degree of health system decentralisation. Interestingly, only one respondent directly raised the resource-constrained nature of their countries as a potential constraint to the implementation of commitments within partnerships.

Political legitimacy, or recognition of the partner’s scope of work and the resultant ‘sphere of influence’, was seen as important, especially given the limited power (discussed above) of some MOHPs within the MOH. In South Africa there was concern that the project should fit within the wider political context, and the project should have legitimacy to be involved in policy issues, as reflected by one MOHP:

"the mandate comes from the ruling party, parliament, minister carries the mandate, and develops policies to address those. So what if for example the project comprises its members from a different to ruling party, who do not have the same mandate as where we are at…"

The distribution of authority between the different levels (national, province and district) was especially important in South Africa and Ghana where the project worked at different levels. Furthermore, in Ghana – in contrast with the other countries - a split between the MOH and Ghana Health Service appeared to have added another level in partnership arrangements.

The perceived lack of consistent commitment across health system levels, potentially resulting from a lack of ownership at all levels, was seen as negatively affecting project implementation. From a partnership perspective, some respondents reflected on the need for the MOHPs to communicate better with other health system levels. Other respondents raised the possibility of establishing multilevel partnership arrangements (between RPs, national-level MOHPs and local administration) to strengthen ownership and commitment at all levels.

### Perceived effects of partnership at different levels

Respondents were asked what effects of partnerships, if any, they could distinguish for themselves as individuals or their institutions. The RPs from Ghana and South Africa suggested that it is methodologically difficult to distinguish the specific effects of partnership as a process, from the wider effects of the MHaPP research project. For example, the greater visibility of mental health as a policy issue, identified by the respondents, could be attributed to partnerships between RPs and MOHPs but could also be a result of MHaPP research. For a similar reason, no distinction was identified between the intended and unintended effects of partnership.

No negative effects of partnerships were identified by respondents. The perceptions of positive effects of partnerships cover the individual, organisational and contextual levels, as set out next.

The most frequently reported partnership effect on RPs was better knowledge of the MOH approaches and working principles, particularly emphasised in Ghana and South Africa.

MOHPs described other effects on individuals, such as enhanced research skills, joint publications and the development of further research proposals. However, despite various thematic capacity strengthening workshops (one of MHaPP’s objectives), attended by both RPs and MOHPs, some MOHPs felt that some of their individual expectations were not met, because:

"…only… research officers always were taken for courses and… the ministry of health officials were…[involved] in… management meeting[s] and not involved in… [research] training."

At the same time some RPs stated that MOHPs had many other commitments and, therefore, did not dedicate sufficient time to the MHaPP research and capacity strengthening activities.

The main organisation-level effect was strengthened collaboration between the MOHPs and RPs. The ability of the MHaPP to jointly identify priorities for interventions at the beginning of Phase 2 was referred to by most respondents as reflecting greater appreciation of each other’s needs. The Zambian MOHP also reflected on the strengthened working relationships between the different MOH Units involved in the project. This could be the result of the more turbulent partnership processes requiring heightened consensus from a wider range of MOH officials. However, it may also reflect the limited relative powers of individuals within the Zambian MOH.

The perceived effects at the wider context level included greater visibility of mental health as a policy issue, improved mental health research in the country, a better overview of the mental health situation and enhanced recognition of mental health issues on the policy agenda.

### Lessons learned from the partnership experience: views of respondents

Three broad lessons can be identified from the above experiences for improving the first two stages of research-policy partnership processes: establishing and supporting & adjusting.

First, all respondents reflected that it is important to develop an agreed set of objectives, to make those objectives clear to all parties and use this as a benchmark for interaction. This can be an important milestone in establishing effective partnerships in the future.

Second, most respondents, especially RPs, emphasised the importance of working hard to maintain dialogue through effective communication, resolving emerging problems and, where appropriate, ensuring personal compatibility of individuals, as reflected by one RP:

"Getting an understanding of the politics, politics of relationships, and politics of negotiating skills and then also advocacy skills, those are the sort of things, and also on the position of the cultural context, in which people operate"

Last, an earlier section identified that both RPs and MOHPs saw the need to recognise each other’s constraints at both individual (expectations and interests) and organisational (structure, decision procedures) levels. The principles of mutual respect and trust were seen as fundamental in this recognition and in ensuring adequate support in maintaining effective relations between parties.

The absence of clear implications for dissolving partnerships from the above findings may either reflect the willingness of partners to continue collaborating in the future or may suggest that research-policy partnerships are long-term relationships with less clear dissolution stages.

## Discussion

The partnership models in all study countries reflect the shift reported in the literature from the two communities’ model towards the linkage and exchange model of partnerships [35]. However, our findings suggest that although researchers and policy-makers worked together within this project, they still represent the two relatively different ‘worlds’ affecting their expectations from, and involvement in, partnerships. We discuss this further in this section.

Three broad components were identified in the conceptual framework (influencing factors, partnership processes and effects of partnership), to which we now return.

Our findings suggest that, within **processes of partnerships**, the interrelationships between partners may experience particularly positive or negative stages. Some of these may reflect the dynamics of building effective relationships as described by Tuckman [44] but the length, and extent of those, positive or negative stages may be the product of compromise, patience and persistence on all sides.

The actual roles and responsibilities of the partners may be different to those in the formal document as was the case in Zambia. Exploring the reasons for these differences lies outside the scope of this study, though the very fact that differences occur suggests a possible need for measures to monitor the implementation of Memoranda of Understanding, or similar documents, in partnerships.

Differing approaches to partnerships are distinguished in the literature, with coordination being the most comprehensive form potentially involving joint planning and implementation of activities and sharing of resources [42,43]. The partnerships in all four countries can be described as lying between cooperation and collaboration; greater coordination of resources (staff and finance) might be the next step in the evolution of the relationships [2].

The mechanisms for conflict resolution appear important in ensuring effectiveness of partnerships. The existence of procedures for conflict resolution depends on the degree of formalisation of partnership arrangements [58]. In MHaPP, the mechanisms for conflict resolution were spelt out in documents such as the research contract and memoranda of understanding. Where partnership arrangements are less formalised, clear mechanisms and processes for effective resolution of disagreements and potential conflicts may also be needed.

The absence, in responses, of references to the Phase of dissolving the partnership may reflect the stage of the project at the time of data collection i.e. before the formal end of MHaPP. It may also suggest that dissolution of partnerships may either occur much later in the process (i.e. partnerships may continue beyond the project life, for example for academic publications) or does not happen at all and successful partnerships are re-formed within future initiatives.

Our findings, consistent with literature, suggest that it is important in designing research-policy partnerships to take account of **key influences affecting effectiveness of partnership** at individual, organisation and contextual/system levels [28,41,45]. This includes recognising the different relationships between the factors at different levels. An example of the latter from this project is related to the concept of collaborative capacity, which comprises skills and expertise of individuals combined with organisational power of these individuals within their institutions or wider system.

Although partners may be attracted by the concept of collaborating towards a common goal, there are different motives for establishing research-policy partnerships – their recognition is an important element of successful partnerships of this nature [28]. Within this project the main reason for establishing partnerships within MHaPP was the need to successfully implement the research project – which also contributed to the dominance of RPs in setting the research agenda and being at the forefront of project decision-making. Researchers can play a more constant role within research-policy partnerships – as was the case in our study – whereas policy-makers’ involvement can vary from being a formal supporter to a responsive audience and to an integral partner [45]. Whether MOH-initiated research-policy partnerships can be equally successful in achieving their objectives and ensuring high-quality research remains open.

It is important to recognise and address, where possible, the different expectations at individual, organisational and systems levels to achieve the optimal and sustainable engagement of all parties and agreeing to the partnership objectives. For example, we anticipated identification of the monetary benefits to individuals and organisations as clear expectations from partnerships. The absence of references to the expectations of monetary benefits may indicate that non-monetary gains (such as improved skills and expertise) are valued more in research-policy partnership. At the same time, although sensitive issues such as monetary gains are often not disclosed as was the case in our study, these may need to be recognised and appropriately managed in motivating involvement of partners in resource-constrained settings.

Clarity on roles and responsibilities of individuals and organisations appears to be critical in ensuring effectiveness of partnerships [1,40,45]. This could be achieved through formalisation of partnership arrangements in a document such as a Memorandum of Understanding, as was used in MHaPP. However, less formal agreements may be appropriate in some contexts - for example, where the MOH may be wary of signing formal documents – though it is important to ensure everyone’s commitment to the partnership goal and objectives.

In our study we specifically focused on MOHP-RP relationships. We recognise, however, that other levels of relationships, such as North–South researcher or MOHP-MOHP interactions, may affect the in-country partnerships. The involvement of the Consortium Coordinator in the research-policy partnership in Zambia is one example where other levels can have a significant impact. It suggests that it is important to recognise these further levels of interaction and, where appropriate, take advantage of these opportunities for enhancing effectiveness of partnerships.

As for the **effects of partnerships**, researchers and policy-makers may have different views about partnership, which influence their involvement in research-policy partnerships [59]. The perception of unmet research-related capacity development expectations of some MOHPs is an example where fulfilling individual expectations, despite their busy schedules, could have contributed to MOHPs being more satisfied with effects of partnerships and providing more inputs towards research.

Effects of partnerships can go beyond a particular initiative, as illustrated by the identification of some contextual effects in our study. It is important to recognise, however, the methodological challenge of attributing the above benefits to a single initiative [8] – in our case MHaPP partnerships - and the need to recognise the changes and influences within the wider country contexts.

Partnerships can potentially have negative effects at different levels. For example, Buse and Harmer identify seven possible negative effects of Global Public-Private Partnerships on national health systems, including skewed national priorities, inappropriate incentives for engaging in partnership and insufficient resources to support partnerships [4] and negative effects can exist within multi-partner research consortia [2]. At a country level, Balloch and Taylor distinguish among others the potential overlaps in service provision resulting from different priorities, different lines of accountability and potential disagreements over management hierarchies and concerns with organisational self-preservation [40]. Although no negative effects of partnerships were explicitly identified in our study, the absence of references to negative effects may either indicate the conceptual difficulty in attributing the negative effects to partnerships (for example, increased workload) or reflect the respondents’ overall satisfaction with the partnerships. The absence of references to negative effects may also be a reflection of social desirability bias i.e. the tendency of respondents to answer questions in a manner that will be viewed favourably by others which typically results in over-reporting positive and/or under-reporting negative effects or behaviour.

Although our primary focus was on partnership processes, we recognised the inevitable overlaps with wider effects of partnerships. In our study we identified effects of partnerships on two broad issues, which are usually seen as the ultimate purposes of research-policy partnerships: whether partnerships helped to improve research quality and added value to GRIPP processes [9,39]. In relation to effects of partnerships on research quality and its use in policy and practice, in many instances the MOHPs’ involvement was perceived as ensuring political support, though some respondents reflected that MOHPs were also involved in conceptualisation and implementation of research. The joint identification of Phase 2 interventions is one example where a shared direction of research was informed by MOHP participation, and may have contributed to MOHP willingness to support the project and to eventually align the research with the MOH priorities. As for the added value of partnerships to GRIPP processes, our findings indicate that the interaction between researchers and policy-makers can facilitate this process, as suggested in the literature [60-63].

Four potential limitations and strengths of this study can be distinguished. First, the study relied on a relatively small number of interviews. However, the sample size for qualitative studies is usually not required to be large, and, our respondents included the lead individuals from both MOHPs and RPs, who were at the forefront of partnerships and thus possessed the detailed knowledge of partnership processes.

Second, as a result of staff changes, some respondents had not been involved in the initiation of the partnership. However, all respondents had knowledge of historical developments of partnerships.

Third, the authorship for this paper includes University of Leeds researchers and those study respondents who contributed to the writing of the paper. The strength of this approach is that a) University of Leeds researchers had detailed knowledge of the research and partnerships while maintaining ‘outsider independence’ by not being directly involved in MOHP-RP relations in the study countries (though we recognise the University of Leeds, being a project partner, had a stake in the success of the partnerships and, therefore may not be complete ‘outsiders’) and b) the authorship complies with the general requirements for contributions to academic publications. However, we recognise that this approach may have influenced the responses given in interviews, and interpretation of the data. Furthermore, during the project the role of lead partners may have shifted from an “outsider “to an “insider” perspective providing access to the information about partnership processes at a country level and contributing to the possible bias. To minimise this, we triangulated findings between different respondent types (MOHP and RPs) and validated our interpretation with respondents.

Last, we recognise that some effects may be difficult to ascertain before dissolution of partnerships. However, the issues related to feasibility of such a study (such as recall bias and access to respondents after the end of partnership) on the whole outweigh the limitations of our approach.

## Conclusions

Recognition of the importance of, and addressing where appropriate, the relationships between researchers and decision-makers in research-policy partnerships can add value to research projects. The conceptual framework developed for this study can be used to understand, design and assess partnerships at their different stages, including their processes. Our study suggests five major implications for setting, maintaining and evaluating future research-policy partnerships.

First, it is important to have a common understanding of mutually-agreed goals and objectives of research-policy partnerships. Ideally these should correspond to the expectations of all parties from partnership, both at individual and organisational levels.

Second, it is important to give attention to the processes of initiating and maintaining research-policy partnerships, based on clear roles, responsibilities, expertise and commitment of both parties at different levels (individual, organisational).

Third, it is important in designing, maintaining and evaluating partnership processes to take account of factors affecting the effectiveness of partnership at individual, organisation and contextual/system levels. These same factors are interrelated and can facilitate or constrain partnerships.

Fourth, although partnerships are often established for a specific purpose such as carrying out a particular project, the effects of partnership can go beyond a particular initiative. It is important, therefore, to recognise the long-term benefits or costs even though many may not be easily identifiable and justifiable within relatively short-term programmes.

Last, we recognise the methodological difficulties in distinguishing the effects of partnership processes from the effects of research in the content area. This is especially important in judging the success of partnerships and distinguishing whether the results/outputs of the project are a reflection of the partnership processes or the nature of the research issue.

This study attempted to contribute to better understanding of processes of research-policy partnerships in the four African countries through developing the conceptual framework and exploring the processes of research-policy partnerships in the four study countries. Further research is needed to test the proposed conceptual framework in other programmes and contexts of different countries.

## Competing interests

All authors were involved in the implementation of Mental Health and Poverty Project. TM, MO, AG and PB were members of the University of Leeds team. CL, AOA and VD were involved, as Research Partners, in research-policy partnerships in South Africa and Ghana. All authors have no further competing interests.

## Authors’ contributions

TM conceived the study, led the study design, data collection, analysis and interpretation of the data and drafting the paper; MO, AG and PB participated in the study design, data collection, analysis and interpretation of the data and drafting the paper; CL, AOA and VD participated in the study design, interpretation and validation of the data and drafting the paper. CL, AOA and VD were involved in partnerships within the respective countries and all authors were involved in the MHaPP project. All authors read and approved the final manuscript.
